# Molecular Surveillance of Carbapenem-Resistant Gram-Negative Bacteria in Liver Transplant Candidates

**DOI:** 10.3389/fmicb.2021.791574

**Published:** 2021-11-22

**Authors:** Tilman G. Schultze, Philip G. Ferstl, David Villinger, Michael Hogardt, Wolf O. Bechstein, Stephan Göttig, Thomas A. Wichelhaus, Stefan Zeuzem, Jonel Trebicka, Oliver Waidmann, Martin-Walter Welker, Volkhard A. J. Kempf

**Affiliations:** ^1^Institute of Medical Microbiology and Infection Control, University Hospital Frankfurt, Frankfurt, Germany; ^2^University Center for Infectious Diseases, University Hospital Frankfurt, Frankfurt, Germany; ^3^University Center of Competence for Infection Control of the State of Hesse, Frankfurt, Germany; ^4^Division of Gastroenterology and Hepatology, Department for Internal Medicine, Goethe University Frankfurt, Frankfurt, Germany; ^5^Department of General and Visceral Surgery, Goethe University Frankfurt, Frankfurt, Germany

**Keywords:** carbapenem resistance, liver transplantation, transmission, whole-genome sequencing, infection control, antimicrobial stewardship

## Abstract

**Background:** Carbapenem-resistant Gram-negative bacteria (CRGN) cause life-threatening infections due to limited antimicrobial treatment options. The occurrence of CRGN is often linked to hospitalization and antimicrobial treatment but remains incompletely understood. CRGN are common in patients with severe illness (e.g., liver transplantation patients). Using whole-genome sequencing (WGS), we aimed to elucidate the evolution of CRGN in this vulnerable cohort and to reconstruct potential transmission routes.

**Methods:** From 351 patients evaluated for liver transplantation, 18 CRGN isolates (from 17 patients) were analyzed. Using WGS and bioinformatic analysis, genotypes and phylogenetic relationships were explored. Potential epidemiological links were assessed by analysis of patient charts.

**Results:** Carbapenem-resistant (CR) *Klebsiella pneumoniae* (*n*=9) and CR *Pseudomonas aeruginosa* (*n*=7) were the predominating pathogens. *In silico* analysis revealed that 14/18 CRGN did not harbor carbapenemase-coding genes, whereas in 4/18 CRGN, carbapenemases (VIM-1, VIM-2, OXA-232, and OXA-72) were detected. Among all isolates, there was no evidence of plasmid transfer-mediated carbapenem resistance. A close phylogenetic relatedness was found for three *K. pneumoniae* isolates. Although no epidemiological context was comprehensible for the CRGN isolates, evidence was found that the isolates resulted of a transmission of a carbapenem-susceptible ancestor before individual radiation into CRGN.

**Conclusion:** The integrative epidemiological study reveals a high diversity of CRGN in liver cirrhosis patients. Mutation of carbapenem-susceptible ancestors appears to be the dominant way of CR acquisition rather than in-hospital transmission of CRGN or carbapenemase-encoding genetic elements. This study underlines the need to avoid transmission of carbapenem-susceptible ancestors in vulnerable patient cohorts.

## Introduction

Liver transplantation (LT) is the only curative treatment for liver cirrhosis, but donor organs are scarce and therefore allocated at a late stage of the disease (Tacke et al., 2016; Adam et al., 2018). Patients on the waiting list for LT are prone to acute decompensation (AD), acute-on-chronic liver failure (ACLF), and hospitalization including application of broad-spectrum antimicrobials due to infections which are closely connected to AD and constitute the most frequent etiology of ACLF, meaning that cirrhotic patients frequently undergo antimicrobial therapy (Fernández et al., 2017; Trebicka et al., 2020). The prevalence of multidrug-resistant organisms (MDRO) in these patients is still rapidly increasing worldwide (Fernández et al., 2012, 2019; Salerno et al., 2017; Piano et al., 2019). Therapeutic options are further limited in case of infection with carbapenem-resistant Gram-negative bacteria (CRGN), often leading to clinical deterioration and death (Ferstl et al., 2017, 2018; Fernández et al., 2019). To prevent transmissions, implementation of infection control measures and an active surveillance of CRGN strains in transplantation centers are crucial (Geladari et al., 2017). Since patients evaluated for LT are regularly screened for CRGN at our center, we reasoned that this vulnerable population might be well-suited for the study of mechanisms leading to carbapenem resistance (CR).

CRGN carriage is detected by screening algorithms (e.g., from rectal swabs) using standard cultivation techniques, species identification, and antimicrobial susceptibility testing methods (Tomczyk et al., 2019). However, the exact genetic resistance determinants and transmission routes often remain unknown. CRGN can be distinguished in carbapenemase-producing (CP) and non-carbapenemase-producing (non-CP) bacteria. The first group harbors carbapenem-hydrolyzing β-lactamases. These isolates represent the most important hygiene threat (Nordmann et al., 2012; Sahuquillo-Arce, 2015) and account for approximately 10% of CRGN cases in patients with liver disease (Ferstl et al., 2017). The group of non-CP CRGN consists of a broad variety of bacterial isolates without expressing a defined carbapenemase. CR in this group is often the result of overexpression of β-lactamases, such as AmpC cephalosporinases or extended spectrum β-lactamases (ESBL) in conjunction with increased efflux or impermeability, for example, due to porin loss (Köhler et al., 1999; Rodríguez-Martínez et al., 2009; Sahuquillo-Arce, 2015; Goodman et al., 2016; Hamzaoui et al., 2018). Various possible explanations for CRGN occurrence exist: (i) CR might be an evolutionary result of mutations leading to loss of porins and/or overexpression of resistance genes in commensal bacteria; this process is triggered by the administration of broad-spectrum antimicrobials and subsequent antimicrobial selection pressure (Sommer et al., 2009; Meletis et al., 2014; Goodman et al., 2016; Iredell et al., 2016). (ii) CR can be acquired by so-called “plasmid hospitalism” which might be the result of a horizontal interspecies gene transfer (Göttig et al., 2015) and, (iii), maybe most important, CRGN can be transmitted directly from patient to patient *via*, for example, shortcomings in infection control measures. Here, we report on the WGS analysis of 18 CRGN isolates obtained from 24 detected CRGN isolates (from 23 patients) while screening of 351 patients evaluated for liver transplantation (Ferstl et al., 2021) over a period of 10years.

## Materials and Methods

### Patients and Ethical Approval

Among 351 patients who were subsequently evaluated and listed for LT at a tertiary German liver care unit between 2008 and 2018, 18 CRGN strains were obtained and further analyzed. All patients had undergone at least one initial screening for any MDRO and had been subjected to further screenings in case of MDRO positivity, as described before. Patients remained in the study until death or last follow-up. Detailed description of screening procedure and a definition of included species are given in the Supplementary Material. Study approval was obtained by the local Ethics Committee for Medical Research of the Medical Faculty, University Hospital, Goethe University, Frankfurt am Main, in accordance with the 1975 Declaration of Helsinki prior to research (file number 268/13). Informed consent was obtained upon study inclusion, and the database was pseudo-anonymized. This study was approved by the local Ethics Committee (November 14, 2018) and was conducted within legal requirements given by German Infection Protection Law (IfSG), in particular §§13, 23 IfSG.

### DNA Sequencing

All bacterial isolates from this study were obtained from our internal culture collection reaching back to the year 2009. Isolates were plated on MacConkey agar (Oxoid), incubated overnight at 37°C, and transferred into PBS solution. DNA isolation was carried out using the smart DNA prep (m) kit (Analytic Jena, Germany) following the manufacturers protocol for Gram-negative bacteria. To enhance bacterial lysis, a pre-lysis treatment with Innuprep bacterial lysis booster (Analytic Jena) was utilized for all samples. Prior to library preparation, purity of all isolated DNA samples was assessed using a Nanodrop spectrometer (Thermo Scientific, Wilmington, United States) and gel electrophoresis. DNA concentration was assessed using a Qubit 2.0 fluorometer (Life technologies, Carlsbad, United States). Library preparation and sequencing were performed by a commercial service provider (Novogene, Cambridge, UK). A detailed description of the method is given in the Supplementary Material.

### Processing of Reads and *de novo* Assembly

For raw read filtering, adapter sequences and reads with more than 10% of indeterminable bases (“N”s) and reads with a low quality (below Q5) for more than 50% of their length were removed. Remaining partial adapter sequences were trimmed using cutadapt (Martin, 2011) version 2.5 on read pairs. All isolates were assembled using unicycler v0.4.8-beta (Wick et al., 2017). Assembly statistics were assessed using a custom script under R version 3.4.4 utilizing the package seqinr (Charif and Lobry, 2007). To be considered as a successful assembly, the total assembled length had to be in line with the expectations (*Klebsiella pneumoniae*: 5.0 to 6.0Mb; *Pseudomonas aeruginosa*: 6.0 to 7.5Mb). In addition, the smallest amount of contigs with a cumulative size equal or bigger than half the genome size (L50) had to be lower than 20 and the respective amount to cover 90% of the genome (L90) had to be below 40.

### Multilocus Sequence Typing and Phylogenetic Analysis

The seven-gene multilocus sequence type for each isolate was determined by running MLST version 2.18.0^1^ on all assembled genomes using PubMLST^2^ (Jolley and Maiden, 2010) database as reference. Prokka (Seemann, 2014) version 1.14.6 was used to perform a whole-genome annotation for all *de novo* assemblies. Resulting gene feature files were used as input for Roary (Page et al., 2015) version 3.13.0 in order to calculate core and accessory genome for the two groups of interest (*P. aeruginosa* and *K. pneumoniae*). For this step, the default 95% identity threshold was applied. Nucleotide alignments of the core genome were used to construct a maximum likelihood tree with fasttree (Price et al., 2010) v2.1.10 using a general time reversible model. To access support of the nodes, 100 random bootstrap replicates were performed. Besides, snp-dists v0.7.0^3^ was used to calculate a matrix of single nucleotide differences in the core genome for all isolates of *K. pneumoniae* or *P. aeruginosa*, respectively. As a mean to visualize similarity of isolates on a whole-genome level, gene presence-absence plots were generated utilizing a community-contributed python script.^4^

### *In silico* Analysis of Antimicrobial Resistance Genes, Porin Sequences, and Plasmids

Identification of antimicrobial resistance genes was carried out by running abricate version 0.9.8^5^ on the assemblies against CARD (Jia et al., 2017) as reference. In cases where several hits were found for one resistance gene, only the best hit was considered. Sequences of porin genes (*ompK35* and *ompK36* for *K. pneumoniae* isolates or *oprD* for *P. aeruginosa* isolates) were obtained from *de novo* assemblies, including a flanking region of 1,000bp on each side. If a contig border disrupted the porin gene or its flanking region, the sequence was manually reconstructed. All sequences were confirmed by aligning the respective paired-end read files with bowtie 2 (Langmead and Salzberg, 2012) version 2.3.4.1 and checking the alignments for mismatches or unaligned ends using CLC Genomics Workbench 12.0.2 (Qiagen, Aarhus, Denmark).

To identify contigs that derive from plasmids, a prediction using RFPlasmid was carried out. To this end, the web service^6^ was utilized selecting the species-specific model “*Enterobacteriaceae*” for *K. pneumoniae* isolates (*n*=9) and the *E. cloacae* isolate (*n*=1), “*Pseudomonas*” for *P. aeruginosa* isolates (*n*=7), and “Metagenomics mode” for the *A. baumannii* isolate (*n*=1). Typing of plasmids is described in detail in the Supplementary Material.

### Analysis of Patient Data Records for Deciphering of Potential Epidemiological Links

To assess potential epidemiological links (e.g., *via* proximity of CRGN patients), attributable to clusters of phylogeny, potential time and room overlaps at outpatient consultations and inpatient stays were retrospectively analyzed. This included (i) determination of concurrent stays on normal wards, intermediate care and intensive care units including admission and discharge dates as well as length of stay, (ii) detection of potential overlaps within any in-house facilities (endoscopy, ultrasound, radiology, surgical theatre) as documented by date and time of procedure, and (iii) assessing the documentation of outpatient consultations. Of note, occupancy of patient rooms was not analyzed further since patients with CRGN which were found to be closely related by phylogenetic analysis had never been admitted to the same ward at the same time.

## Results

### Prevalence of CRGN in LT Patients

**Figure 1:**
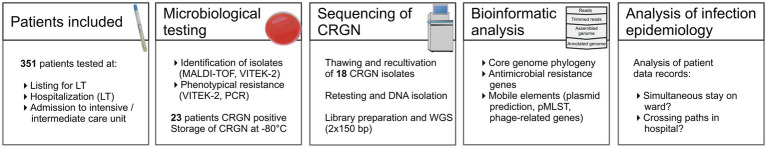
Workflow of sampling, clinical microbiology, sequencing, and data analysis.

From 2008 to 2018, 351 patients evaluated and listed for LT had undergone MDRO screening according to the in-house hygiene plan (Ferstl et al., 2021). Among these 351 patients, 24 CRGN were identified in 23 patients, including one patient with the detection of two CRGN (*K. pneumoniae* and *P. aeruginosa*). Of those, 18 CRGN isolates were recovered from the in-house isolate collection, which were used for further analysis (*K. pneumoniae*: *n*=9, *P. aeruginosa*: *n*=7, *Acinetobacter baumannii*: *n*=1, and *Enterobacter cloacae*: *n*=1). The workflow consisting of sampling, conventional clinical microbiology, WGS, and bioinformatics is depicted schematically in Figure 1, and infection sites are given in Table 1.

**Table 1:** CRGN bacteria analyzed in this study. of note, all but three specimens (A_008, A_016, and A_018) were also found in rectal swabs, meaning that 14/17 patients (82%) were intestinally colonized.

Isolate	Species	Timespan from first detection to infection (d)	Infection site	Timespan from first detection to last infection (d)	Timespan from first detection to death (d)
A_001	*K. pneumoniae*	n/a	n/a	n/a	7
A_002	*K. pneumoniae*	77	tracheal secretion	77	83
A_003	*K. pneumoniae*	0	abscess drainage	0	137
A_004	*K. pneumoniae*	8	urine, catheter-associated, abscess, surgical site, bronchial lavage, ascites	71	86
A_005	*K. pneumoniae*	n/a	n/a	n/a	21
A_006	*P. aeruginosa*	0	blood	65	72
A_007	*P. aeruginosa*	0	decubitus	0	X
A_008	*P. aeruginosa*	0	bronchial lavage	7	18
A_009	*E. cloacae*	n/a	n/a	n/a	52
A_010	*K. pneumoniae*	72	decubitus, wound	116	254
A_011	*K. pneumoniae*	n/a	n/a	n/a	100
A_012	*P. aeruginosa*	792	bile	1,205	2,118
A_013	*P. aeruginosa*	33	decubitus, wound	44	182
A_014	*K. pneumoniae*	0	biliary drainage	0	X
A_015	*P. aeruginosa*	69	blood	69	69
A_016	*A. baumannii*	n/a	n/a	n/a	X
A_017	*K. pneumoniae*	20	urine, surgical site, bile, blood	1,089	X
A_018	*P. aeruginosa*	0	wound, biliary drainage, bronchial lavage	103	114

*n/a,not available as patients were only colonized. X,no time span to death (surviving patients)*.

### Timeline Analysis

**Figure 2:**
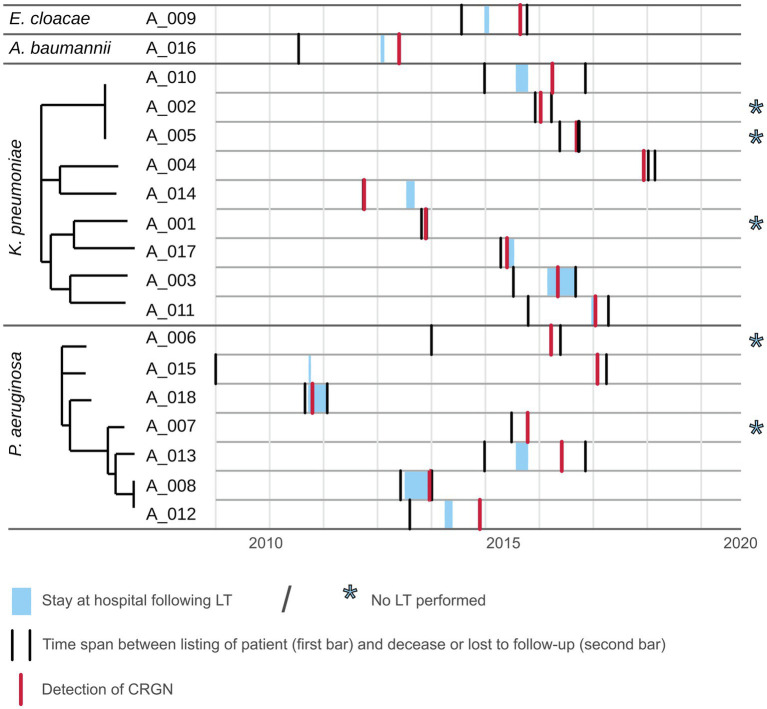
Timeline plot for patients with CRGN listed for LT. Depicted as gray areas are the time span from listing for LT until loss to follow-up or decease for each patient for which a CRGN was detected. Light blue areas within represent the stay at Frankfurt University hospital following LT. Red bar marks the initial CRGN detection.

Median (min; max) time from listing to CRGN detection was 479 (40, 3,006) days. Twelve patients underwent LT of whom nine died. CRGN were detected in two patients before LT (one fatality) and in ten patients after LT (eight fatalities). Among those five patients who did not undergo LT, four patients died (Table 1). At closing of data acquisition, 14/17 CRGN patients have died. Fatalities were distributed almost equally among patients with *K. pneumoniae* (*n*=5) and *P. aeruginosa* (*n*=6; Table 2). Patients with closely related isolates (A_002, A_005 and A_010) were never admitted simultaneously to a particular ward nor had they crossed paths within in-house facilities and services (e.g., radiology and endoscopy) on the same day (see Figure 2).

**Table 2:** Clinical courses of CRGN patients with or without LT.

	All patients (*n* =17)	LT patients (*n* =12)	Non-LT patients (*n* =5)
Timespan from LT listing to CRGN detection	223 (−35; 2,937)	397 (−35; 2,937)	124 (33; 920)
Timespan from LT listing to LT	n/a	251(24; 715)	n/a
Timespan from LT listing to death	479 (40; 3,006)	504 (51; 3,006)	136 (40; 992)
Timespan from LT to CRGN detection	n/a	191 (−76; 2,222)	n/a
Timespan from CRGN detection to death	83 (7; 2,118)	100 (18; 2,118)	46(7; 83)

*Values (days) are given as medians with range (min; max). n/a, not applicable for patients of this group*.

### Phylogeny and Whole-Genome Analysis of Potentially Related Isolates

Phylogenetic analyses based on single nucleotide polymorphisms (SNP) in the core genome revealed a cluster of three *K. pneumoniae* isolates (A_002, A_005 and A_010). Two *P. aeruginosa* isolates (A_008 and A_012) clustered as well; however, the difference of more than 50 SNPs was considered too high for direct patient-to-patient transmission and was therefore not further analyzed. When comparing the sequence of the 4,071 core genes of the three clustering *K. pneumoniae* isolates, A_002 and A_005 differed by only 6 SNPs or 22 SNPs for the comparison of A_002 and A_010.

**Figure 3:**
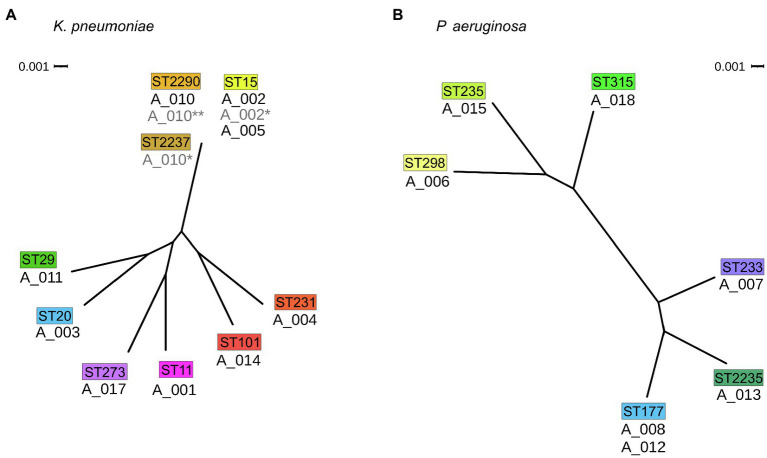
Phylogenetic relatedness of isolates. **(A)** Phylogenetic tree of *K. pneumoniae* isolates and **(B)** *P. aeruginosa* isolates of this study both in radial view. The corresponding MLST types are drawn above the isolates in colored boxes. Scale bar: phylogenetic distance.

To control whether related carbapenem-susceptible isolates from these three patients were previously obtained, three contemplable isolates were sequenced and analyzed *a posteriori*. These were an isolate sampled 28days after A_002 (termed A_002^*^), one isolate obtained 203days prior to A_010 (termed A_010^*^), and one isolate sampled 103days after A_010 (termed A_010^**^). When included in the phylogenetic analysis, all three isolates clustered with A_002, A_005 and A_010 (see Figure 3).

Whole-genome analysis of the isolates A_002, A_005, and A_010 revealed differences in several loci. Most notably, isolate A_005 lacks a 12kb large region resulting in a loss of *mgrB* known to be associated with colistin resistance (Cannatelli et al., 2014). In line, A_005 was the only colistin-resistant isolate (MIC >8mg/ml) in phenotypical resistance testing. Besides, A_005 uniquely possesses a cassette of four genes including a SHV-type ESBL gene.

### *In silico* Characterization of Antimicrobial Resistance Genes

#### Carbapenemases

**Figure 4:**
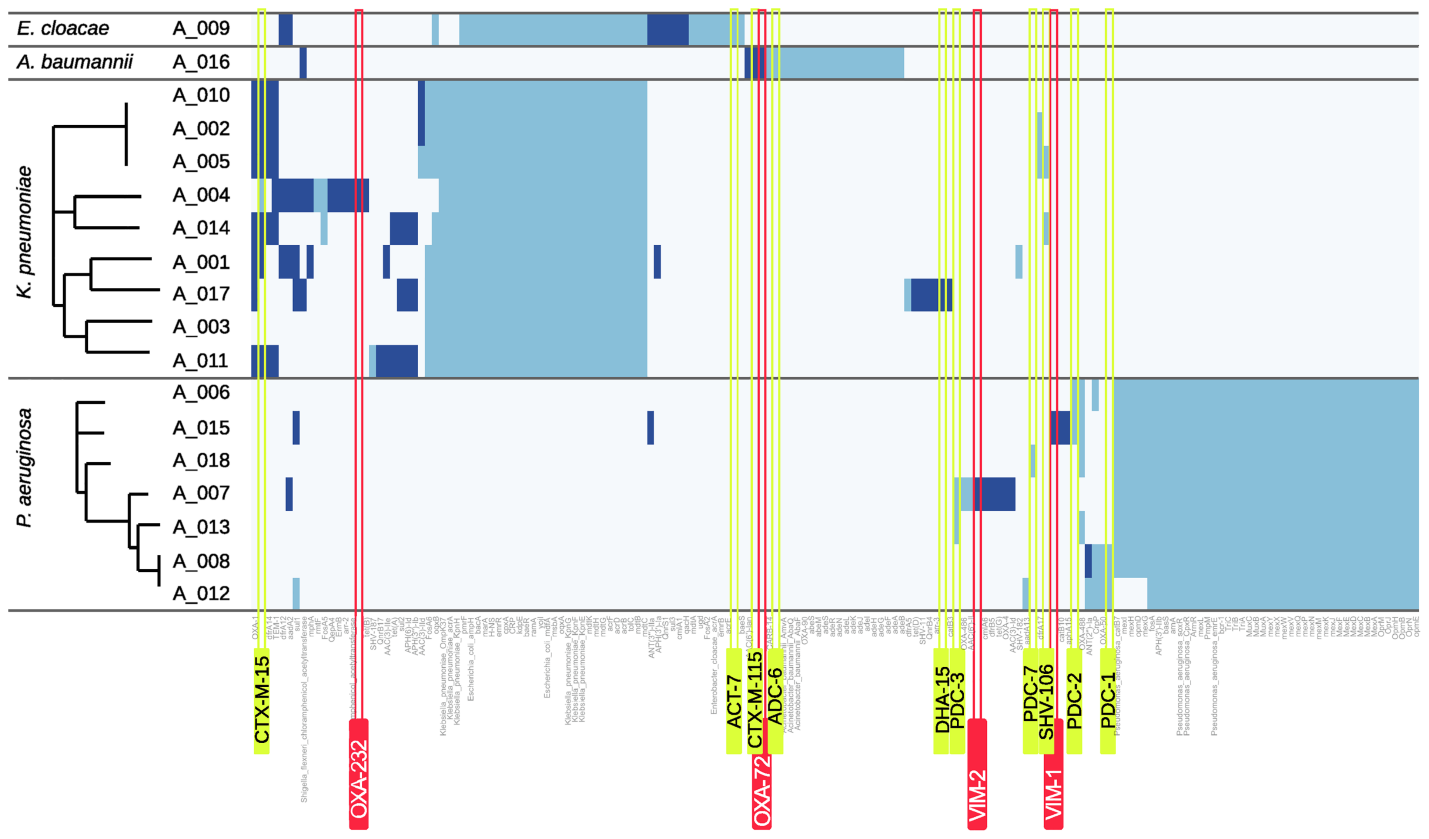
Heatmap of resistance genes. Hits for resistance genes are shown as blue bars. Dark blue indicates that the respective gene is predicted to be plasmid-related whereas light blue indicates a chromosomal origin. Carbapenemase genes are highlighted by red and *ampC* or ESBL genes by yellow boxes.

A mass screening for antimicrobial resistance genes against the comprehensive antibiotic resistance database (CARD) was carried out and for each resistance gene the location (plasmid or chromosome) was predicted (Figure 4). In four isolates, carbapenem-hydrolyzing β-lactamase genes were detectable: A_004 (*K. pneumoniae*) harbors an OXA-232 gene belonging to the family of OXA-48-like carbapenemases. A_016 (*A. baumannii*) carries a copy of OXA-72 belonging to the family of OXA-24-like carbapenemases. Two *P. aeruginosa* isolates were found to harbor two Verona integron metallo-β-lactamase genes (A_015: VIM-1, A_007: VIM-2).

#### Non-carbapenemase Determinants

Only isolate A_017 harbors an *ampC* gene with DHA-15 which was identified to be plasmid-encoded.

In contrast, several ESBL genes were found in the analyzed genomes. *A. baumannii* isolate A_016 scores a plasmid-located hit for the ESBL gene *bla*_CTX-M-115_ and the *K. pneumoniae* isolates A_005 and A_014 both harbor the ESBL gene *bla*_SHV-106_. However, *bla*_CTX-M-15_ was found most frequently in *K. pneumoniae* isolates (7/9 isolates). To investigate whether plasmid-mediated spread especially for these genes occurred within the group of our *K. pneumoniae* isolates, all respective isolates were analyzed using Plasmidfinder and pMLST. Similar plasmid allele types were found only for the three isolates A_002, A_005, and A_010 (data not shown).

#### Genes Coding for Porins

**Figure 5:**
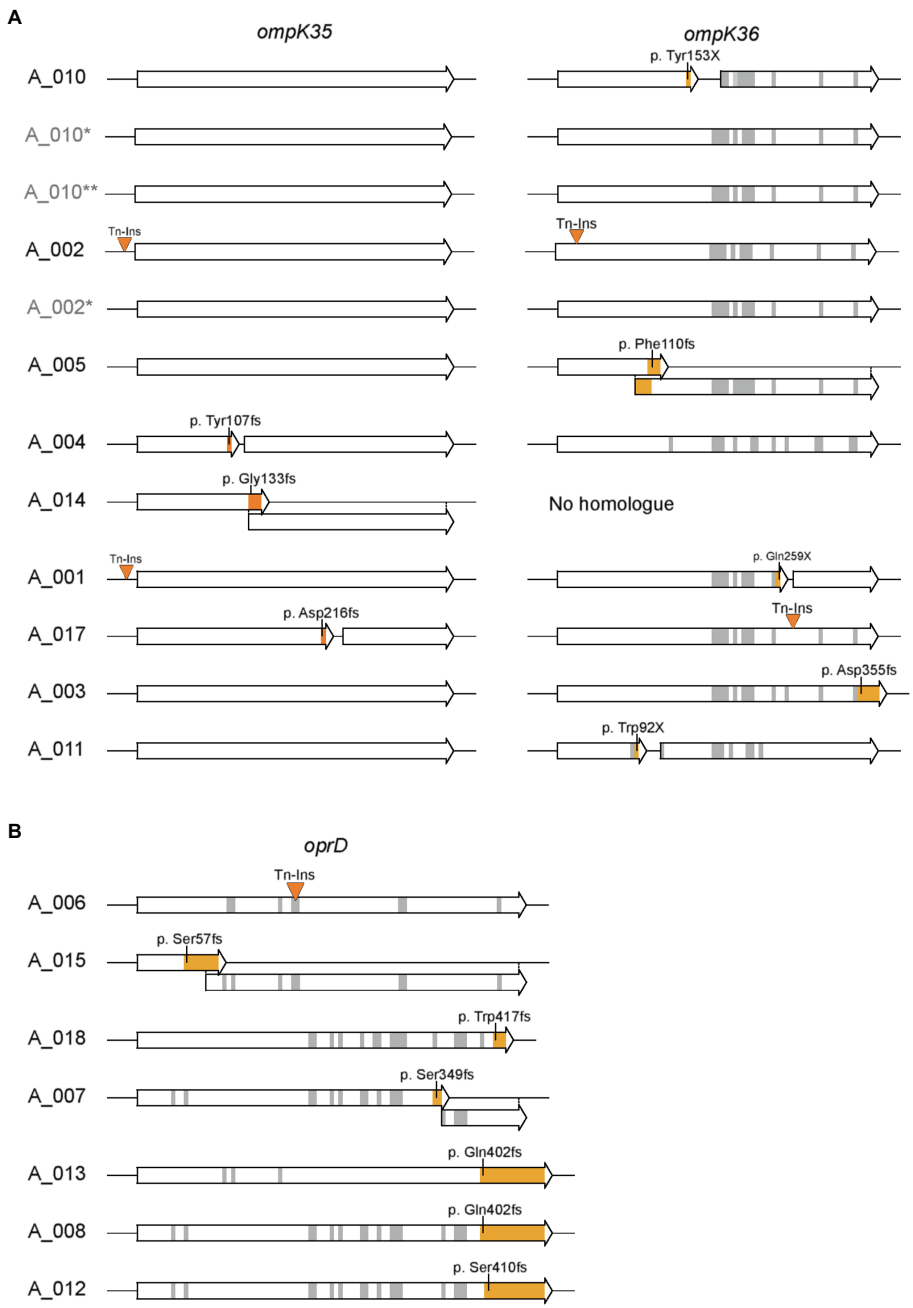
Sequence analysis of porins involved in carbapenem resistance. **(A)** Sequences of *ompK35* and *ompK36* for *K. pneumoniae* isolates and **(B)** sequences of *oprD* for *P. aeruginosa* isolates are shown. Insertions of transposons and mutations leading to a frameshift or insertion of a premature stop codon are highlighted in orange. Gray areas represent other amino acid changing mutations. Amino acid positions and sequence refer to *ompK35* and *ompK36* of *K. pneumoniae* ATCC43816 (CP009208.1) or *oprD* of *P. aeruginosa* PAO1, respectively. Isolates labeled in light gray (A_002^*^, A_010^*^, and A_010^**^) indicate carbapenem-sensitive isolates from the respective patient obtained prior or post the CRGN isolate.

For porins known to contribute to carbapenem resistance in case of mutations or loss, the deduced sequence was analyzed (Köhler et al., 1999; Rodríguez-Martínez et al., 2009; Hamzaoui et al., 2018). These were OmpK35 and OmpK36 for the nine *K. pneumoniae* isolates and OprD for the seven *P. aeruginosa* isolates. Results highlight that both *P. aeruginosa* and *K. pneumoniae* isolates accumulated a variety of unique mutations leading to a frameshift or an insertion of a premature stop codon (Figure 5). In addition, transposon insertions were found 15bp upstream of *ompK35* for two isolates, within *ompK36* for two isolates, and within *oprD* for one isolate. Remarkably, the three closely related isolates A_002, A_005, and A_010 exhibit different mutations: While transposon insertions were observed for A_002 in both *ompK35* and *ompK36*, a frameshift mutation was found in *ompK36* in A_005 and a mutation resulting in a premature gene stop was found in A_010. In case these isolates derived from a common ancestor or source, these isolates acquired subsequent mutations of the porin genes individually. This notion is supported by fact that neither of the carbapenem-susceptible isolates A_002^*^, A_010^*^ nor A_010^**^ exhibit any of these mutations.

## Discussion

Colonization with CRGN is associated with repeated hospitalization, sepsis, and increased mortality in patients with advanced liver disease (Ferstl et al., 2017, 2021). Therefore, it is of utmost importance to avoid transmission of CRGN to patients that are awaiting or underwent LT.

The results of our retrospective study provide no evidence for a gross transmission of CRGN bacteria or CR genes/plasmids mediating resistance determinants from patient to patient for the herein analyzed patient cohort. These conclusions can be drawn out of the observations that (i) most bacterial isolates were identified as phylogenetically unrelated, (ii) carbapenemases (OXA-232, VIM-1, VIM-2, and OXA-72) were each found in not more than one isolate, respectively, and (iii) there was no evidence for plasmid-mediated spread of resistance determinants mediating CR. These observations contrast with studies from other centers where a certain sequence type and or carbapenemase type were dominantly witnessed (Clancy et al., 2013; Karampatakis et al., 2017; Macesic et al., 2018). Dissemination of certain sequence types carrying carbapenemases is known to vary drastically depending on region (Nordmann et al., 2011; Logan and Weinstein, 2017). Therefore, our data indicate that an individual assessment and surveillance of CRGN might be needed for each center.

In our cohort, non-CP CRGN isolates were the predominant resistance determinants accounting for 14 out of 18 isolates. For non-CP CRGN, CR is often mediated by ESBL and/or AmpC genes in conjunction with porin deficiencies (Köhler et al., 1999; Rodríguez-Martínez et al., 2009; Hamzaoui et al., 2018). Porin-mediated resistance cannot be easily transferred between pathogens and might even come with a fitness cost for the respective isolate (Nordmann et al., 2012; Wong et al., 2019); however, the underlying mutations can arise over time and there is evidence that non-CP-mediated resistances are more likely to arise under antimicrobial pressure (Goodman et al., 2016). Obviously, this is especially troublesome as antimicrobials are indispensable in the treatment of patients with decompensated cirrhosis and ACLF.

Our results strongly suggest that three of the analyzed CRGN derive from a carbapenem-susceptible ancestor. Therefore, strict infection control measures for patients at risk even at those time points before the detection of CR bacteria are required. Such measures might avoid the spread of certain bacterial ancestor strains with a high genetic plasticity for evolving CR resistance which is of special importance as spread of such carbapenem-susceptible pathogens will mostly not be noticed. In case of the three isolate of our study, careful analysis of patient data records did not uncover any potential transmission event. For identification of such events, it is necessary to trace all contact points of patients to the healthcare system. For instance, transmission of CRGN *via* an outpatient clinic was reported (Klein et al., 2021) and it is highly likely that a part of LT patients is under a shared medical supervision. Patients might have crossed at healthcare facilities, such as general practitioners, primary or secondary hospitals, or even nursing and rehabilitation facilities. As relevant outpatient histories could not be retrieved due to the retrospective design and demise of almost all patients, the only possibility here was to reconstruct any patient-to-patient encounters inside of our hospital but defined crossing points were not detected.

Although the in-house hygiene plan with strict screening algorithms for MDRO allows quick and reliable CRGN detection in cirrhotic inpatients, acquisition of carbapenem-susceptible common ancestors might occur unregistered as screening for antimicrobial-susceptible bacteria is not done routinely. Tracking down such strains, however, requires a comprehensive study design including all participating healthcare providers and is also of high microbiological complexity as there is no easy screening algorithm for carbapenem-susceptible pathogens carrying an intrinsic susceptibility for development of carbapenem resistance.

**Figure 6:**
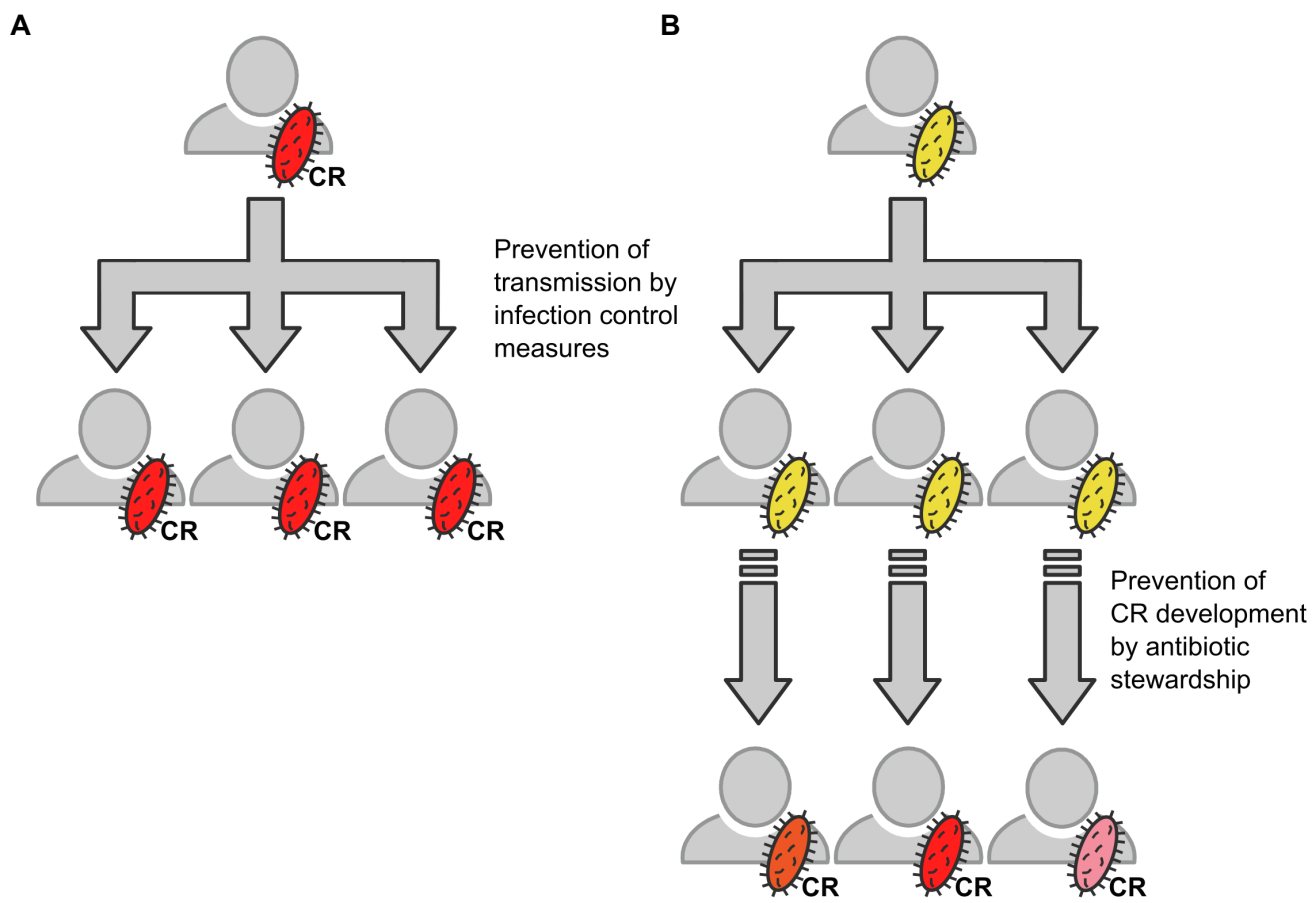
Schematic draft of the herein described ways resulting in acquisition of CR bacteria and the respective countermeasures. **(A)** Direct transmission of CRGN bacteria (schematically depicted in red), for example, from patient to patient *via* shortcomings in infection control. **(B)** Transmission of a carbapenem-sensitive ancestor bacterium (schematically depicted in yellow) and subsequent individual development of carbapenem resistance (schematically depicted by different red color shapes) under antimicrobial pressure, for example, *via* inappropriate antimicrobial therapy.

In summary, rather than transmission of CRGN or CR determinants within our cohort, our data support a model according to which CRGN evolve under antimicrobial pressure. Transmission of carbapenem-susceptible ancestor pathogens may be an early step within this process (see Figure 6). This implicates that (i) the transmission of potential ancestors should be avoided by strict infection control measures even before occurrence of CR bacteria and (ii) the wise use of antimicrobials should be pursued especially in patients with carbapenem-susceptible multidrug-resistant Gram-negative bacteria. Antimicrobial stewardship efficiently reduces antimicrobial burden in, for example, oncologic patients (Pillinger et al., 2020), yet its value in patients with decompensated cirrhosis and ACLF still needs to be demonstrated. While hospital hygiene stays an integral part in the care of cirrhosis and LT, further strategies to avoid acquisition of CR clearly need to be implemented. To this end, prospective studies including close-mesh microbiological screening and a surveillance of pathogens using WGS are urgently warranted.

## Data Availability Statement

The datasets presented in this study can be found in online repositories. The names of the repository/repositories and accession number(s) can be found at: https://www.ncbi.nlm.nih.gov/sra/SRP327397/.

## Ethics Statement

The studies involving human participants were reviewed and approved by Ethics Committee of the University Hospital Frankfurt am Main, Germany. The patients/participants provided their written informed consent to participate in this study.

## Author Contributions

VK, M-WW, TS, and PF designed the study. DV, MH, SG, and TW were involved in microbiological screening procedures, sample collection, and sample preparation. TS performed bioinformatic analysis. PF, WB, SZ, OW, and M-WW were responsible for clinical patient care and patient charts. VK, TS, and PF wrote the manuscript. All authors contributed to the article and approved the submitted version.

## Funding

This work was supported by the State of Hesse, Germany (“Hessisches Universitäres Kompetenzzentrum für Krankenhaushygiene” and the LOEWE-Center “ACLF-I, project P5”). The funders had no role in study design, data collection and analysis, decision to publish, or preparation of the manuscript.

## Conflict of Interest

The authors declare that the research was conducted in the absence of any commercial or financial relationships that could be construed as a potential conflict of interest.

## Publisher’s Note

All claims expressed in this article are solely those of the authors and do not necessarily represent those of their affiliated organizations, or those of the publisher, the editors and the reviewers. Any product that may be evaluated in this article, or claim that may be made by its manufacturer, is not guaranteed or endorsed by the publisher.

## Footnotes

^1^ https://github.com/tseemann/mlst

^2^ https://pubmlst.org/

^3^ https://github.com/tseemann/

^4^ https://raw.githubusercontent.com/sanger-pathogens/Roary/master/contrib/roary_plots/roary_plots.py

^5^ https://github.com/tseemann/abricate

^6^ http://klif.uu.nl/rfplasmid/

## Abbreviations

CRGN: Carbapenem-resistant gram-negative bacteria
WGS: Whole-genome sequencing
CR: Carbapenem-resistant
LT: Liver transplantation
AD: Acute decompensation
ACLF: Acute-on-chronic liver failure
MDRO: Multidrug-resistant organisms
CP: Carbapenemase-producing
ESBL: Extended spectrum Β-Lactamases
IfSG: German infection protection law (“Infektionsschutzgesetz”)
SNP: Single nucleotide polymorphisms
