# Supplementary material for: Molecular Surveillance of Carbapenem-Resistant Gram-Negative Bacteria in Liver Transplant Candidates
Source: Front Microbiol. 2021 Nov 22;12:791574. doi: 10.3389/fmicb.2021.791574 (PMC8645865; doi:10.3389/fmicb.2021.791574)
Supplement: Supplementary file 1 [file Data_Sheet_1.DOCX]

Supplementary Material

# Supplementary information

## Screening procedure and definitions

According to German infection law implementation of an infection control protocol in order to prevent the transmission of infective agents (e.g., CRGN) is mandatorily required. At the University hospital Frankfurt, this legal requirement by the German Infection Protection Law (IfSG) as well as the recommendations of the German Commission for Hospital Hygiene and Infection Prevention (KRINKO) are entirely fulfilled. Therefore, patients reporting defined risk factors, e.g., arriving from high-prevalence countries, as well as patients admitted to any intensive/intermediate care unit are screened for MDRO including CRGN at the day of admittance as reported earlier (Reinheimer et al., 2016). Isolates referred to as CRGN are a subgroup of multidrug-resistant Gram-negative bacteria that, besides ESBL phenotype (*Enterobacterales*) or resistance against piperacillin, ceftazidime and fluoroquinolones (*P. aeruginosa*), carry additional acquired resistance to carbapenems. Patients were defined as “colonized” if CRGNs were detected in nasal, rectal or pharyngeal swabs or “infected” if CRGN were detected in wounds or sterile compartments.

## Detection of CRGN and molecular resistance analysis

Species identification of presumed MDRO and antibiotic susceptibility testing were performed as previously described using standard clinical microbiology methods(Reinheimer et al., 2016). Matrix-assisted-laser desorption ionization-time of flight analysis (MALDI–TOF; VITEK MS) and VITEK-2 (bioMérieux, Nürtingen, Germany) were used to identify Gram-negative species from cultures. Antibiotic susceptibility testing was carried out according to the recommendations of the Clinical and Laboratory Standards Institute (CLSI; until 2018) and EUCAST (from 2019) by using VITEK-2, antibiotic gradient tests (Liofilchem, Roseto degli Abruzzi, Italy; Etest®, bioMérieux, Nürtingen, Germany) or agar diffusion (Oxoid, Wesel, Germany). Carbapenemase-encoding genes were detected via polymerase chain reaction analysis and subsequent sequencing from carbapenem-resistant Enterobacterales including the *bla* genes for carbapenemases OXA–48 like, KPC, NDM, VIM and IMP as well as OXA–23, OXA–24, and OXA– 58 for *A. baumannii**.* All laboratory testing was performed under strict quality-controlled criteria (laboratory accreditation according to ISO 15189:2007 standards; certificate number D–ML–13102–01–00).

## Library preparation and sequencing

DNA was randomly fragmented by sonication aiming for a size of 350 bp, ends of fragments were repaired and A-tailed to facilitate subsequent ligation of sequencing adapters. Sequences within these adapters were used for PCR amplification. Purification of library fragments was carried out using Ampure XP beads (Beckman Coulter, High Wycombe, UK). Finally, all libraries were pooled and sequenced on a NovaSeq 6000 flow cell (Illumina, San Diego, CA) in a multiplexed manner that yields 5 to 7 million reads per isolate. As sequencing strategy, a paired-end approach of 2x150bp was chosen.

## Detection of CRGN and molecular resistance analysis

To characterize the putative plasmids of *K. pneumoniae* isolates, Plasmidfinder 2.1 (v 2.0.1, database version 2020-07-13; accessible via https://cge.cbs.dtu.dk/services/PlasmidFinder/) and pMLST 2.0 (v 0.1.0, database version 2020-10-05, accessible via https://cge.cbs.dtu.dk/services/pMLST/) was run on *de novo* assemblies using allele sequences and profiles obtained from PubMLST ([http://pubmlst.org](http://pubmlst.org/)).

# References

Reinheimer, C., Kempf, V. A. J., Göttig, S., Hogardt, M., Wichelhaus, T. A., O’Rourke, F., et al. (2016). Multidrug-resistant organisms detected in refugee patients admitted to a University Hospital, Germany June‒December 2015. *Eurosurveillance* 21, 30110. doi:10.2807/1560-7917.ES.2016.21.2.30110.
